# Health Behaviors Associated With Overweight and Obesity Among Physicians

**DOI:** 10.1002/osp4.70129

**Published:** 2026-03-06

**Authors:** Dana Kagansky, Racheli Magnezi, Shlomit Koren, Matan Elkan, Orna Tal

**Affiliations:** ^1^ Shamir Medical Center (Assaf Harofeh) Zerifin Israel; ^2^ Endocrine Institute Department of Otolaryngology Shamir Medical Center (Assaf Harofeh) Zerifin Israel; ^3^ Department of Management Health Systems Management Program Bar Ilan University Ramat Gan Israel; ^4^ Internal Medicine A Shamir Medical Center (Assaf Harofeh) Zerifin Israel

**Keywords:** health behavior, obesity, organizational wellness, physician well‐being, workplace health

## Abstract

**Background:**

Overweight and obesity among physicians are increasingly recognized as relevant to workforce health, quality of care, and the credibility of lifestyle counseling delivered to patients. Physicians may face constraints that affect dietary patterns, physical activity, and daily routine, behaviors directly related to BMI. However, data linking specific lifestyle behaviors to BMI in this population remain limited.

**Objective:**

To examine associations between self‐reported health behaviors and BMI among physicians and outline implications for workplace wellness.

**Methods:**

Cross‐sectional online survey of physicians using the 26‐item Healthy Lifestyle and Personal Control Questionnaire (HLPCQ). Participants were categorized into normal weight (BMI < 25) and overweight or obesity (BMI ≥ 25) groups based on their BMI. Group differences and multivariable logistic regression were applied.

**Results:**

Of 200 respondents, 144 complete cases were analyzed; 54.2% had a BMI < 25. Higher BMI was associated with lower adherence to health‐promoting behaviors, notably Dietary Harm Avoidance (*p* < 0.01), Daily Routine (*p* = 0.05), and Organized Physical Exercise (*p* = 0.03). Regression analysis identified Dietary Harm Avoidance and Organized Physical Exercise as protective factors against overweight and obesity (*p* < 0.01). Physicians in surgical specialties reported lower health behavior scores than non‐surgical peers, particularly in Daily Routine (*p* < 0.01) and in total lifestyle scores (*p* = 0.01).

**Conclusions:**

Specific lifestyle behaviors, particularly dietary harm avoidance and organized physical activity, were associated with BMI among physicians. These associations may help inform future research on modifiable behavioral factors related to overweight and obesity among physicians.

## 1 Introduction

The profound impact of lifestyle choices on overweight and obesity has led preventive medicine to increasingly emphasize health promotion and disease prevention [1]. Healthcare professionals play a pivotal role in these efforts, both as providers of care and as exemplars for healthy living, as patients may interpret alignment between physicians' personal behaviors and their clinical advice as a signal of authenticity and credibility [2, 3, 4, 5]. While physicians have traditionally been regarded as role models, public trust in healthcare professionals has fluctuated in recent years, particularly during and following the COVID‐19 pandemic in some settings [6, 7, 8]. Nevertheless, within the clinical encounter, physicians' personal health behaviors may still influence their credibility, communication, and patient engagement in lifestyle‐related counseling [9, 10, 11].

However, despite their professional knowledge and the expectation that they should exemplify healthy living, many healthcare professionals struggle to adhere to the very lifestyle recommendations they advocate for their patients [9]. Long working hours, burnout, and sleep deprivation are significant barriers that hinder their ability to maintain healthy behaviors, leading to overweight and obesity rates among healthcare professionals that are comparable to, or even higher than, those of the general population. This is particularly evident among nurses and physicians who face greater challenges [12, 13]. In this context, workplace‐related constraints such as limited time for regular meals, irregular schedules affecting daily routine, and reduced opportunities for organized physical activity may directly influence specific lifestyle domains assessed by the HLPCQ. Importantly, physicians' health behaviors represent an intersection between individual well‐being and professional functioning, as the same occupational constraints that shape physicians' lifestyle choices may be associated with both health and their engagement with lifestyle‐related counseling in clinical practice [14].

Consistent with this perspective, previous studies have shown that physicians' personal health behaviors, including weight status, diet, and physical activity, are associated with their professional engagement in lifestyle counseling and patient care [15, 16]. Physicians with healthier personal behaviors are more likely to counsel patients on weight management and physical activity and are perceived as more credible role models in health promotion [4]. However, much of the existing literature has focused on individual behaviors or weight status alone, with less attention to broader patterns of lifestyle behaviors and their relationship with overweight and obesity among physicians.

Understanding the health behaviors of physicians and their impact on being overweight or obese is critical, as they serve as role models and advisors for healthy living within the healthcare system. The present study used the Healthy Lifestyle and Personal Control Questionnaire (HLPCQ) to explore the relationship between self‐reported health behaviors and BMI among physicians [17]. The HLPCQ was designed to assess self‐empowerment through daily health‐related activities, including diet, exercise, routines, and social support. By focusing on physicians, this study highlights the unique perspective of medical professionals who face distinct challenges and responsibilities that may influence their health behaviors and consequently their BMI.

## 2 Methods

### 2.1 Study Design and Participants

This cross‐sectional study explored the relationship between self‐reported health behaviors and BMI among physicians. Participants were practicing physicians working across a range of clinical settings, including hospital‐based and community‐based practice. Data were collected through an anonymous online questionnaire, which included demographic information, BMI data, and HLPCQ responses. Medical specialty was self‐reported by participants as part of the demographic section of the questionnaire. The questionnaires were distributed to approximately 600 physicians via email in September 2024, using mailing lists specifically targeting physicians. Responses were collected through the end of November 2024. Only questionnaires with complete data for BMI and all HLPCQ items were included in the final analysis. Questionnaires with missing or incomplete responses were excluded. Participants self‐reported their height and weight, which were used to calculate BMI. They were categorized into normal weight (BMI < 25) and overweight or obese (BMI ≥ 25). Obesity was not analyzed as a separate group due to sample size considerations and to maintain adequate statistical power for the planned analyses. BMI was analyzed categorically to align with established clinical thresholds and to facilitate clinically meaningful interpretation of overweight and obesity. The study protocol was approved by the Institutional Review Board of Shamir Medical Center, and informed consent was implied by voluntary completion of the anonymous survey.

### 2.2 Healthy Lifestyle and Personal Control Questionnaire (HLPCQ)

The respondents were asked to complete the HLPCQ questionnaire. This is a 26‐item tool that assesses the frequency of adopting various lifestyle habits across five domains, using a 4‐point Likert scale (1 = Never or rarely, 2 = Sometimes, 3 = Often, and 4 = Always). The subscales are: Dietary Healthy Choices (questions 1–7; score range 7–28), which evaluates the frequency of healthy dietary practices; Dietary Harm Avoidance (questions 8–11; score range 4–16), which measures behaviors aimed at avoiding dietary risks; Daily Routine (questions 12–19; score range 8–32), which assesses consistency in daily routine, including meal and sleep schedules; Organized Physical Exercise (questions 20–21; score range 2–8), which measures engagement in structured exercise activities; and Social and Mental Balance (questions 22–26; score range 5–20), which evaluates social support practices and mental well‐being. Total HLPCQ scores ranged from 26 to 104, with higher scores indicating healthier lifestyle behaviors. The questionnaire was used to capture lifestyle patterns potentially correlating with BMI among physicians.

The full HLPCQ is provided in Supporting Information S1.

### 2.3 Analysis by Medical Specialty

In addition to the primary analysis, participants were classified into surgical and non‐surgical specialties, and HLPCQ domain scores were compared between these groups. Medical specialty was self‐reported by participants and subsequently classified according to the primary clinical role. Non‐surgical specialties included internal medicine, family medicine, and psychiatry, whereas surgical specialties included general surgery, gynecology, orthopedics, and other surgical disciplines, including smaller procedural fields such as ophthalmology and otolaryngology.

### 2.4 Statistical Analysis

A 95% confidence interval was evaluated to estimate parameter precision and uncertainty. All *p*‐values were two‐tailed, with *p*‐values ≤ 0.05 considered statistically significant. For categorical variables, the Chi‐square test or Fisher's exact test was applied based on expected cell frequencies. For continuous variables, distributional assumptions were assessed and appropriate parametric (Student's *t*‐test) or non‐parametric (Mann–Whitney *U*) tests were selected accordingly. Logistic regression was used for multivariable analyses, with each lifestyle domain and the total HLPCQ score entered separately into the model. All models were adjusted a priori for age, sex, marital status, religiosity and area of residence, which were selected as standard sociodemographic covariates commonly collected in epidemiological studies and consequently associated with BMI. Covariates were included regardless of statistical significance in univariate analyses to allow appropriate adjustment for potential confounding. Statistical analysis and data visualization were performed using Python (v3.10) and Microsoft Excel (v2401).

## 3 Ethics Approval and Consent to Participate

This study was conducted under the approval of the Ethics Committee of Assaf Harofeh Medical Center (Shamir Medical Center), in compliance with the Declaration of Helsinki (approval number: 0104‐24‐ASF). Participation was voluntary and informed consent was implied through completion of the anonymous, electronic questionnaire.

## 4 Results

The questionnaire was distributed to 200 physicians, of whom 144 completed full answers. Table 1 compares the sociodemographic characteristics of the study population according to the BMI category (< 25 vs. ≥ 25 kg/m^2^). The mean age was 42.4 ± 10.2 years with no significant difference in the age between the two groups. Women were more prevalent in the BMI < 25 group compared with the BMI ≥ 25 group (75.6% vs. 54.7%, *p* = 0.01). There were no significant differences between BMI categories in marital status, area of residence and religiosity level, except for a borderline difference in the traditional group.

**TABLE 1:** Demographic characteristics of participants by BMI category (normal < 25 vs. overweight and obesity ≥ 25).

Variable^a^	Total (*n* = 144)	BMI < 25 (*n* = 78)	BMI ≥ 25 (*n* = 66)	*p*‐value
Age, years (m ± SD)	42.4 ± 10.2	40.9 ± 8.4	44.3 ± 11.8	0.26
Female^b^	94 (66.20%)	59 (75.64%)	35 (54.69%)	0.01
Marital status				
Single	15 (10.42%)	9 (11.54%)	6 (9.09%)	0.84
In a relationship	125 (86.81%)	67 (85.90%)	58 (87.88%)	0.92
Divorced	2 (1.39%)	1 (1.28%)	1 (1.52%)	1.00
Widowed	2 (1.39%)	1 (1.28%)	1 (1.52%)	1.00
Religiosity				
Secular	104 (72.22%)	58 (74.36%)	46 (69.70%)	0.66
Traditional	7 (4.86%)	1 (1.28%)	6 (9.09%)	0.05
Religious	32 (22.22%)	18 (23.08%)	14 (21.21%)	0.95
Ultra‐orthodox	1 (0.69%)	1 (1.28%)	0 (0%)	1.00
Residence				
Urban	112 (77.78%)	61 (78.21%)	51 (77.27%)	1.00
Suburban	16 (11.11%)	10 (12.82%)	6 (9.09%)	0.66
Rural	16 (11.11%)	7 (8.97%)	9 (13.64%)	0.53

Abbreviations: BMI, body mass index; SD, standard deviation.

^a^ Data are presented as mean ± standard deviation or *n* (%).

^b^ Two (1.4%) participants did not report sex and were not included in this analysis.

HLPCQ domain scores by BMI category are presented in Table 2. The total HLPCQ score was significantly higher in participants with BMI < 25 compared with those with BMI ≥ 25 (62.14 ± 10.74 vs. 57.53 ± 8.49, *p* = 0.01). Participants with BMI < 25 scored higher than those with BMI ≥ 25 in the dietary harm avoidance and organized physical exercise subscales (*p* < 0.01 and *p* = 0.03, respectively), with a borderline difference observed in the daily routine domain (*p* = 0.05).

**TABLE 2:** Healthy Lifestyle and Personal Control Questionnaire domain scores by BMI category.

Variable^a^	All (*n* = 144)	BMI < 25 (*n* = 78)	BMI ≥ 25 (*n* = 66)	Mean differences (95% CI)	*p*‐value
Dietary healthy choices	15.50 ± 3.29	15.50 ± 3.16	15.50 ± 3.46	0.00 (−1.10, 1.10)	0.75
Dietary harm avoidance	9.79 ± 2.34	10.54 ± 2.39	8.91 ± 1.95	1.63 (0.91, 2.34)	< 0.01
Daily routine	17.72 ± 5.37	18.51 ± 5.51	16.77 ± 5.09	1.74 (−0.01, 3.49)	0.05
Organized physical exercise	4.63 ± 2.17	5.01 ± 2.25	4.18 ± 2.00	0.83 (0.13, 1.53)	0.03
Social and mental balance	12.39 ± 2.49	12.58 ± 2.52	12.17 ± 2.46	0.41 (−0.41, 1.23)	0.48
Total	60.03 ± 10.01	62.14 ± 10.74	57.53 ± 8.49	4.61 (1.44, 7.78)	0.01

Abbreviations: CI, confidence interval; HLPCQ, Healthy Lifestyle and Personal Control Questionnaire; SD, standard deviation.

^a^ Data are presented as mean ± standard deviation.

Comparison of HLPCQ domain scores between surgical and non‐surgical specialties is presented in Table 3. The total HLPCQ score was significantly lower among physicians in surgical specialties compared with those in non‐surgical specialties (55.29 ± 7.64 vs. 60.98 ± 10.19, *p* = 0.01). Among the individual HLPCQ domains, non‐surgical specialties demonstrated higher scores across domains; however, a statistically significant difference was observed only in the daily routine domain (*p* < 0.01).

**TABLE 3:** Comparison of Healthy Lifestyle and Personal Control Questionnaire domain scores between surgical and non‐surgical specialties.

Variable^a^	All (*n* = 144)	Surgical specialties (*n* = 24)	Non‐surgical specialties (*n* = 120)	Mean differences (95% CI)	*p*‐value
Dietary healthy choices	15.50 ± 3.29	15.12 ± 2.59	15.57 ± 3.42	−0.45 (−1.69, 0.79)	0.78
Dietary harm avoidance	9.79 ± 2.34	9.38 ± 1.97	9.88 ± 2.40	−0.50 (−1.43, 0.43)	0.36
Daily routine	17.72 ± 5.37	14.83 ± 4.82	18.29 ± 5.31	−3.46 (−5.68, −1.23)	< 0.01
Organized physical exercise	4.63 ± 2.17	4.33 ± 1.93	4.69 ± 2.22	−0.36 (−1.26, 0.54)	0.51
Social and mental balance	12.39 ± 2.49	11.62 ± 2.32	12.54 ± 2.51	−0.92 (−1.98, 0.15)	0.19
Total	60.03 ± 10.01	55.29 ± 7.64	60.98 ± 10.19	−5.68 (−9.35, −2.02)	0.01

Abbreviations: CI, confidence interval; HLPCQ, Healthy Lifestyle and Personal Control Questionnaire; SD, standard deviation.

^a^ Data are presented as mean ± standard deviation.

Table 4 presents adjusted logistic regression analyses examining the associations between individual HLPCQ domain scores and overweight status (BMI ≥ 25), with each domain adjusted separately for age, sex, marital status, religiosity, and area of residence. Higher scores in dietary harm avoidance, daily routine, and organized physical exercise domains were significantly associated with lower odds of being overweight. In addition, a higher total HLPCQ score was independently associated with reduced odds of being overweight.

**TABLE 4:** Logistic regression analyses of Healthy Lifestyle and Personal Control Questionnaire domains and overall score predicting overweight status.

Variable^a^	Adjusted odds ratio	Confidence interval	*p*‐value
Dietary healthy choices	0.98	0.88–1.10	0.76
Dietary harm avoidance	0.70	0.59–0.83	< 0.01
Daily routine	0.92	0.85–0.99	0.02
Organized physical exercise	0.79	0.67–0.95	0.01
Social and mental balance	1.00	0.86–1.15	0.96
Total	0.94	0.91–0.98	< 0.01

Abbreviations: CI, confidence interval; OR, odds ratio.

^a^ Data are presented as adjusted odds ratios with 95% confidence intervals. Each variable was adjusted separately for age, sex, marital status, religiosity, and area of residence.

Comparisons of HLPCQ subscores between males and females are presented in Supporting Information S1: Tables S1 and S2. There was no significant difference in the total HLPCQ score. Females scored significantly higher than males on the subscale of dietary harm avoidance domain.

## 5 Discussion

Taken together, these findings suggest that physicians' lifestyle behaviors may be shaped by occupational demands, positioning personal well‐being and professional functioning along a shared behavioral continuum rather than as separate domains.

Within this framework, the findings demonstrate that lifestyle behaviors, as measured by the HLPCQ, varied significantly among physicians and were associated with BMI. Physicians with higher BMI consistently reported lower adherence to health‐promoting behaviors, including dietary harm avoidance, daily routine management, and organized physical exercise. This aligns with prior studies showing that work‐related stress, long hours, and physical and mental exhaustion significantly hinder the ability to adopt and maintain healthy habits [18, 19]. These barriers reflect broader organizational and occupational factors within healthcare settings that may influence physicians' ability to adopt and maintain healthy lifestyle behaviors. As originally validated, the HLPCQ is interpreted at both the total score and individual subscale levels, allowing identification of specific lifestyle domains with relatively lower adherence rather than relying on threshold‐based classification.

Physicians in surgical specialties demonstrated significantly lower scores in the Daily Routine domain and the Total score compared to their non‐surgical counterparts, reflecting the unique demands of surgical practice, such as long hours, overnight shifts, and limited opportunities for meals and rest [20]. These demands may disrupt health‐promoting behaviors and contribute to burnout [21, 22, 23]. The observed differences in daily routine and total lifestyle scores are consistent with prior studies describing the demanding schedules and work–life balance challenges characteristic of surgical practice [23, 24]. The regression analysis further underscored the protective role of dietary harm avoidance and organized physical exercise in supporting a healthy BMI among physicians. Dietary harm avoidance and dietary healthy choices capture conceptually distinct aspects of eating behavior, with the former reflecting avoidance of overtly unhealthy options under time pressure, and the latter reflecting proactive and often aspirational behaviors that may be less sensitive to occupational constraints. Interestingly, the lack of significant association in dietary healthy choices and social and mental balance raises questions about their role in this context and warrants further investigation.

This cross‐sectional, single‐center study has several limitations, including limited ability to infer causality and the possibility of reverse causality between lifestyle behaviors and BMI. The reliance on self‐reported height and weight used to calculate BMI may introduce reporting bias. BMI was analyzed categorically rather than as a continuous variable, which may have reduced sensitivity to detect more subtle associations across the BMI spectrum. In addition, the relatively low response rate raises the possibility of selection bias, as respondents may differ from non‐respondents in health awareness or lifestyle behaviors. The small number of physicians in surgical specialties limited the ability to perform more detailed subgroup analyses. Given that multiple lifestyle domains were examined, domain‐specific findings should be interpreted as exploratory. Future studies using objective measures and larger samples may further clarify the associations between lifestyle behaviors and overweight and obesity among physicians.

## 6 Conclusion

This study provides valuable insights into the health behaviors of physicians and associations with BMI, addressing an important issue, given the role healthcare professionals play as societal role models. Physicians are uniquely positioned to influence patient attitudes and behaviors through personal health choices [25]. However, the results highlight the challenges they face in maintaining optimal health habits. These challenges are strongly shaped by workplace conditions and organizational demands.

This study highlights the complex relationship between BMI and health behaviors among physicians, emphasizing the need for systemic support through workplace wellness programs, organizational scheduling policies, and supportive institutional cultures to enable healthcare professionals to adopt and maintain healthier lifestyles. By addressing these challenges through targeted interventions, healthcare systems may foster a healthier and more resilient workforce, better equipped to lead by example and reinforce public trust in health messages. Such efforts align with workplace behavioral health priorities by linking employee well‐being, organizational sustainability, and quality of care.

## Author Contributions

D.K. and O.T. contributed to the concept and design of the study, data acquisition, analysis, and interpretation. D.K. was primarily responsible for the statistical analysis and manuscript drafting. O.T. provided critical supervision and ensured methodological rigor. R.M. contributed to data collection, literature review, and manuscript preparation. S.K. and M.E. were involved in data management, organization, and critical revision of the manuscript for intellectual content. All authors have read and approved the final manuscript.

## Funding

The authors have nothing to report.

## Conflicts of Interest

The authors declare no conflicts of interest.

## Supporting information


Supporting Information S1

## Data Availability

The data collected for this study were obtained through the secure survey platform of Ben‐Gurion University, which ensures full anonymity and confidentiality of participants' responses. In accordance with institutional and ethical guidelines, the data are not publicly available to protect participant privacy. Access to the data may be granted upon reasonable request, subject to ethical approval and compliance with relevant data protection regulations. Further enquiries can be directed to the corresponding author.
