# Supplementary material for: Health Behaviors Associated With Overweight and Obesity Among Physicians
Source: Obes Sci Pract. 2026 Mar 6;12(2):e70129. doi: 10.1002/osp4.70129 (PMC12966352; doi:10.1002/osp4.70129)
Supplement: Supplementary file 1 — Supporting Information S1 [file OSP4-12-e70129-s001.docx]

**Supplementary table S1.** Sex proportions between specialty groups

| **Group** | **Female (n)** | **Male (n)** | **Total (n)** |  |
| --- | --- | --- | --- | --- |
| **Surgical** | 14 | 8 | 22 |  |
| **Non-Surgical** | 80 | 40 | 120 |  |
|  |  |  |  |  |
|  |  |  |  |  |

**Supplementary table S2.** Comparison of Healthy Lifestyle and Personal Control Questionnaire domain scores between males and females

| **Domain** | **Female** | **Male** | **P-value** |
| --- | --- | --- | --- |
| **Dietary Healthy Choices** | 15.56±3.44 | 15.38±3.07 | 0.74 |
| **Dietary Harm Avoidance** | 10.09±2.28 | 9.31±2.36 | 0.04 |
| **Daily Routine** | 17.72±5.44 | 17.85±5.25 | 0.80 |
| **Organized Physical Exercise** | 4.55±2.25 | 4.77±1.99 | 0.51 |
| **Social and Mental Balance** | 12.62±2.37 | 12.1±2.63 | 0.36 |
| **Total** | 60.54±10.2 | 59.42±9.63 | 0.53 |

*Data are presented as mean ± standard deviation
